# Effect of Pharmaceutical Intervention in Pharmacologically Treated Hypertensive Patients—A Cluster-Randomized Clinical Trial: AFPRES-CLM Study

**DOI:** 10.3390/jpm13101484

**Published:** 2023-10-11

**Authors:** Raúl Luque del Moral, Miguel A. Gastelurrutia, Fernando Martinez-Martinez, Julio A. Jacomé, Ana Dago, Blanca Suarez, Narjis Fikri-Benbrahim, Mercé Martí, Cristina Nuñez, Sandra Sierra-Alarcón, Francisco-José Fernandez-Gomez

**Affiliations:** 1 Pharmaceutical Care Research Group, University of Granada, 18011 Granada, Spain; magastelu@gmail.com (M.A.G.); femartin@ugr.es (F.M.-M.); bsuarezluque@gmail.com (B.S.); narjisfikri@yahoo.es (N.F.-B.); 2 Council of Official Associations of Pharmaceutics of Castilla-La Mancha, 45005 Toledo, Spain; 3 Group of Cellular and Molecular Pharmacology, Department of Pharmacology, CEIR Campus Mare Nostrum, University of Murcia, Campus de Ciencias de la Salud, 30120 Murcia, Spain; crisnp@um.es (C.N.); franciscojose.fernandez@um.es (F.-J.F.-G.); 4 Pharmaceutical Care Foundation, 08017 Barcelona, Spain; julioandres444@gmail.com (J.A.J.); anadagom@gmail.com (A.D.); merce.marti@alumni.esade.edu (M.M.); 5 Murcia Research Institute of Health Sciences (IMIB), 30120 Murcia, Spain

**Keywords:** arterial hypertension, community pharmacy care program, outcomes health, pharmaceutical intervention, blood pressure control

## Abstract

Background: Evaluate the effect of a community pharmaceutical intervention on the control of blood pressure in hypertensive patients treated pharmacologically. Methods: A cluster-randomized clinical trial of 6 months was carried out. It was conducted in the Autonomous Community of Castilla-La Mancha (Spain). Sixty-three community pharmacies and 347 patients completed the study. Intervention patients received the community pharmaceutical intervention based on a protocol that addresses the individual needs of each patient related to the control of their blood pressure, which included Health Education, Pharmacotherapy Follow-up and 24 h Ambulatory Blood Pressure Measurement. Control patients received usual care in the community pharmacy. Results: The pharmaceutical intervention resulted in better control of blood pressure (85.8% vs. 66.3% *p* < 0.001), lower use of emergencies (*p* = 0.002) and improvement trends in the physical components of quality of life, measured by SF-36 questionnaire, after 6 months of pharmaceutical intervention. No significant changes were observed for any of these variables in the control group. There were also detected 354 negative medication-related outcomes that were satisfactorily resolved in a 74.9% of the cases and 330 healthcare education interventions and 29 Ambulatory Blood Pressure Monitorings were performed in order to increase adherence to pharmacological treatment and minimize Negative Outcomes associated with Medication and prevent medication-related problems. Conclusions: Community pharmaceutical intervention can increase hypertensive patients with controlled blood pressure, after 6 months, compared with usual care.

## 1. Introduction

Arterial hypertension (AHT) is a worldwide public health problem affecting 30–45% of the general population and increasing markedly at more advanced ages. It is considered to be one of the main cardiovascular risk factors (CVRFs) and is responsible for very high morbidity and mortality [1].

Worldwide, AHT occupies first place in the list of factors associated with the appearance of diseases [2]. The relationship between blood pressure (BP) and the risk of developing cardiovascular disease (CVD) is continuous, consistent and independent of other CVRFs.

The prevalence of AHT in Spain is high; 42.6% of the Spanish adult population is hypertensive and BP is controlled in only 30.0% [3]. This lack of BP control is similar to the situation in other countries in our region of a similar socio-economic level [4]. These figures are considered very discouraging, given the huge healthcare efforts and economic resources invested in controlling this health problem. In terms of absolute mortality, it is estimated that AHT is associated with the deaths of about 40,000 people per year in the Spanish population aged 50 years or more [5].

BP control rates have improved in Spain, though not sufficiently, and this may be related, at least in part, to greater use of antihypertensive treatment, particularly combined therapy [6]. Despite this greater control it is far from optimal, especially in patients with higher cardiovascular risk (CVR) [7].

Poor BP control can be regarded as a current healthcare reality in most countries, and the best way to control this health problem has not been identified. In this context, community pharmacies could be a very important healthcare resource for controlling the hypertensive population [8]. In this context, a per-protocol community pharmaceutical care program, consisting of a multi-component pharmaceutical intervention, could therefore contribute substantially to improving the health results obtained with medications, in humanistic and economic as well as clinical terms [9].

Spanish law, regarding the provision of pharmaceutical care, establishes that pharmacists will be in charge of ensuring the compliance with the recommendations ordered by the doctor responsible for patient prescription. Community pharmacists will cooperate in monitoring the treatment through pharmaceutical care procedures, contributing to ensuring effectiveness and safety. Among the pharmaceutical care processes are pharmacotherapy-related problems such as contraindications, duplications, prescription errors and interactions (special attention to pharmacogenetics). Thus, pharmaceutical care constitutes a safety filter in the detection of these possible incidents and errors with medications. Interestingly, different reports indicate that up to 80% of errors related to the diagnosis, prescription and use of medications could be prevented [10,11,12]. The objective of the AFPRES-CLM study was to determine the impact of a community pharmaceutical care program on the clinical, humanistic and economic outcomes of patients diagnosed with AHT and receiving pharmacological treatment.

## 2. Materials and Methods

### 2.1. Participation of the Patients

Participating pharmacists have developed the AFPRES-CLM program for which they have been properly trained. The participating pharmacies were recruited by open calls inviting participation in the study, through the official professional associations of pharmacists. Every pharmacy participated voluntarily.

Once the community pharmacists performed the first interview and had access to the clinical history, patients diagnosed with and pharmacologically treated for essential AHT, who were literate, in full possession of their mental faculties and willing to be monitored for 6 months, were included. Patients with arrythmia or secondary AHT or who were pregnant were excluded.

The patients participated in the study voluntarily, and for this purpose they were provided with information on the characteristics of the study and signed an informed consent, informing them of the objective of the study, and freely agreed to take part. The patients were free to withdraw from the study and were selected as they entered the community pharmacy, met the inclusion criteria and wanted to participate in the study. Control patients went to the pharmacy twice, while intervention patients went to the pharmacy at least once a month to apply the AFPRES-CLM program. The intervention patients were referred to the doctor, when necessary.

### 2.2. Definitions

AFPRES-CLM is a cluster randomized controlled trial (retrospective registration TCTR20190313004 www.thaiclinicaltrials.org) conforming to CONSORT guidelines [13]. It was conducted in the Autonomous Community of Castilla-La Mancha (Spain) and was approved by the Clinical Research Ethics Committee of the Hospital General Universitario in Ciudad Real (09/2014).

The main study variable was the percentage of patients with controlled BP, defined as systolic BP (SBP) ≤ 140 mm Hg and diastolic BP (DBP) ≤ 90 mm Hg in the general population, SBP ≤ 140 mm Hg and DBP ≤ 85 mm Hg in diabetic patients and SBP ≤ 150 mm Hg and DBP ≤ 90 mm Hg in older people aged over 80 years, as established in the European guidelines for the management of AHT [1].

### 2.3. Design

We designed a community intervention study with a minimum of 80 pharmacies participating, 40 intervening pharmacies and 40 control pharmacies, and a minimum of 480 patients, 240 in the control group (CG) and 240 in the intervention group (IG). Once the pharmacies interested in taking part in the study had been recruited, in each of the five provinces in the region, they were randomly assigned to the CG or the IG and undertook to recruit and monitor 6 patients undergoing antihypertensive treatment for a period of six months (January–June 2015).

One of the most useful methods to identify Negative Outcomes associated with Medication (NOMs) is the classification afforded by the Third Consensus of Granada [14]. Using this categorization, the pharmacists may detect the NOMs divided into necessity (untreated health problem or effect of unnecessary medicine), effectiveness (non-quantitative ineffectiveness or quantitative ineffectiveness) and safety (non-quantitative safety problem or quantitative safety problem) employing pharmacotherapeutic monitoring performed by the Dader method in the community pharmacy [15]. Thus, using this tool pharmacists may take decisions and prevent Medication-Related Problems (MRPs). It is important to note that self-medication, such as having phytotherapy and nutrition supplement, may cause interactions with the current pharmacological treatment. Thus, the pharmacists of the CG received instructions by phone and email about the protocol of the study, in addition to performing the usual pharmaceutical care in the community pharmacy for hypertensive patients treated pharmacologically. These patients made two visits to the pharmacy during their monitoring, at the beginning and at the end of the study, and received the usual assistance in the community pharmacy for a pharmacologically treated hypertensive patient, while those in the IG attended the pharmacy at least one per month to receive a per-protocol, structured pharmaceutical care program for hypertensive patients, consisting of the following activities: (a) Health Education to enhance the patients’ knowledge of their disease, adherence to and rational use of medicines, (b) Pharmacotherapeutic Monitoring (PTM), to identify, prevent and resolve MRPs and NOMs and (c) Twenty-four-hour Ambulatory Blood Pressure Monitoring (ABPM), for uncontrolled patients in whom measurement was indicated [16] who tolerated the device and on whom ABPM had not previously been performed. The measurements were taken with approved and clinically validated automatic devices.

The IG pharmacists received a complementary training program, consisting of monthly clinical sessions, designed to enable them to tackle the various situations arising in patients and to intervene on an individualized basis. This training activity addressed the following subjects: AHT and medications, BP measurement techniques, ABPM, cardio-healthy lifestyle habits, CVR and clinical cases in the AFPRES-CLM program.

At the beginning and end of the follow-up period, in both groups, BP was measured at the pharmacy with approved and clinically validated devices. These devices were accepted by the International Consensus on Arterial Hypertension.

Moreover, registered information was collected on the use of healthcare resources (visits to a primary care doctor, hospital admissions, visits to an emergency department and visits to a specialist) associated with AHT during the 6 months preceding the study period, and quality of life (QL) was measured using the SF-36 questionnaire. This is a generic instrument for measuring health-related QL, with an available version adapted to the Spanish population, appropriate for use in research and clinical practice [17]. If patients in the IG needed to be referred to a doctor, referrals were made in accordance with the criteria agreed between the Spanish Arterial Hypertension Society–Spanish League for Fighting Arterial Hypertension (SEH-LELHA), the Spanish Community Pharmacy Society (SEFAC) and the Pharmaceutical Care Research Group at the University of Granada (GIAF-UGR) [18].

### 2.4. Statistical Analysis

To calculate the sample size we took into account the fact that studies measuring QL require large samples of patients to detect significant changes, since these measurements are generally less sensitive to changes than physiological measurements and a large number of patients is therefore required to detect an impact on QL. In this study, we estimated the sample size on the basis of the QL parameter. We calculated the size of sample needed to make a comparison of two independent means in parallel groups. To analyze the quality of life of hypertensive patients using the SF-36 questionnaire, accepting an alpha risk of 0.05 and a beta risk of 0.20 in a two-sided test, a minimum of 240 subjects was needed in each group to detect a difference of at least 0.2 units, assuming a common standard deviation of 0.7 and having an estimated a loss-to-follow-up rate of 20%. We also made an estimate of the necessary sample size according to the clinical effectiveness parameter (BP control), obtaining a smaller size than the previous calculation. We therefore considered this sample size estimate sufficient for both parameters.

As regards the statistical analysis, the sample was initially described by calculating absolute and relative frequencies for the qualitative variables, and means and standard deviations or medians and percentiles for the quantitative variables, with or without a normal distribution, respectively. Initially, we compared the clinical and demographic variables for each group (intervention or control) by applying hypothesis testing and calculating the corresponding statistical test. To compare percentages, we calculated chi-squared values with Yates’ correction in 2 × 2 tables, correcting them with Fisher’s test if the minimum frequency conditions per cell were not met. When the variable to be compared between the study groups was quantitative, we applied the Student’s *t*-test to compare means for variables with a normal distribution, and in another case the Mann Whitney nonparametric *U* test.

To analyze a possible significant change in these variables derived from intervention, we applied dependent samples tests, as well as generalized linear model repeated measures, to study the changes over time in the clinical and humanistic variables according to the group they belonged to, by calculating Wilks’ lambda statistic.

All statistical analyses were performed using SPSS 18 for Windows (SPSS, Chicago, IL, USA) and *p* ≤ 0.05 was considered statistically significant.

## 3. Results

The study was started with 63 community pharmacies and 388 patients, 174 men (44.8%) and 214 (55.2%) women. Of these, 202 patients (52.1%) belonged to CG and 186 (47.9%) belonged to IG.

In the development of the study there were 41 lost patients. In the CG there were 22 patients lost (3 deaths, 4 patients who changed residence and 15 patients did not go to the pharmacy in the final visit) while in the IG there were 19 lost patients (2 patients died, 6 patients changed residence and 11 patients did not attend scheduled visits at the pharmacy). The AFPRES-CLM program was completed by 63 pharmacies, 32 (50.8%) in the CG and 31 (49.2%) in the IG, with a total of 347 patients, of whom 158 (45.8%) were male and 189 (54.2%) were female.

The mean age of the patients was 67.4 ± 11.5 years. Of the total number of patients, 52.1% belonged to the CG and 47.9% to the IG, and the characteristics of the two groups were comparable, except age (68.5 in the CG vs 66.1 in the IG, (* *p* ≤ 0.05)) (Table 1).

At the end of the monitoring period the percentage of patients with controlled BP was higher in the IG than in the CG (85.8% vs. 66.3%), a statistically significant difference (Table 2).

With regard to QL, we found no significant differences between the groups at the end of the study. In the comparison between before and after the monitoring period, no statistically significant differences in the CG were found in any of the dimensions, whereas in the IG we found a significant improvement in the physical pain and overall physical health dimensions (Table 3).

As for use of healthcare resources, in the final comparison between the groups we found a statistically significant difference in favor of the IG in the use of emergency departments. However, no significant differences were found in hospital admissions or in visits to primary care doctors or specialists (Table 4).

A total of 354 NOMs were detected in patients in the IG. The most frequent NOMs were those in the effectiveness category (61.9%), followed by safety (20.0%) and need (18.1%). The distribution of the various types of MRP is shown in Table 5. A total of 265 MRPs (74.9%) were resolved satisfactorily, of which 177 (66.8%) were resolved by referral to a primary care doctor and 88 (33.2%) were resolved directly by the pharmacist.

The pharmacists performed a total of 330 educational interventions during the course of the program. The most frequent type of intervention was nutritional education. The pharmacists detected 29 patients in whom 24 h ABPM measurement was indicated, and the test was performed. It was considered invalid in 13 cases, as it did not reach the percentage of correct measurements necessary to determine its validity [1]. Of the 16 valid ABPMs, 11 patients were found to have a dipper profile and 5 had an anomalous profile (2 risers, 2 non-dippers and 1 extreme dipper). The patients who underwent ABPM measurement were referred to a primary care doctor for evaluation. Out of the 16 valid ABPMs, the primary care doctor made changes in the treatment of 6 patients (37.5%). Of the group of 29 patients who were given the ABPM test, the BP of 27 (93.0%) was controlled at the end of the monitoring period. The two remaining subjects were considered uncontrolled hypertensive patients relative to the main objective of the study.

## 4. Discussion

Nowadays, the best way to solve AHT is not known. Therefore, a protocolized and individualized community pharmaceutical intervention could be an indispensable ally in the approach to this disease. This justifies the necessary collaboration of the community pharmacist to tackle this health problem according to our knowledge. This is the first study carried out in Spain evaluating clinical, economic and humanistic outcomes of pharmacologically treated hypertensive patients.

Medications are the prime healthcare resource used to solve patients’ health problems. It is noteworthy to distinguish between clinical pharmacology that performs clinical tasks, and pharmaceutical pharmacology that is in charge of tasks related to the therapy by itself, but from the drug side. Some studies point to important changes in the use patterns of antihypertensive drugs during the last few decades, with trends towards increasing use despite the absence of major new developments in the existing pharmacological groups. The use of antihypertensives has increased all over Europe and the increase in Spain is similar to the European mean [19,20]. Therefore, the availability of antihypertensive medications does not seem to be the reason for the lack of BP control and we need to look for other possible causes. The strategic position of community pharmacists can be ideal for improving the management of AHT [21]. A pharmaceutical intervention implemented on a programmed, protocolled, structured and individualized basis, adapted to the particular needs of each patient, could contribute substantially to improving BP control, as well as other clinical, humanistic and economic outcomes of patients with AHT [22]. In this context, the AFPRES-CLM program was developed for hypertensive patients under pharmacological treatment, providing for individualized care in both pharmacological and non-pharmacological areas.

BP control requires synergies and the active participation of the various healthcare professionals involved [23,24]. It is well known that collaboration between doctors and pharmacists improves the rate of BP control [25,26] and the use of medications, and is also economically advantageous for the healthcare system [27]. Adopting these collaborative healthcare strategies could be very effective in reducing CVD, as well as being profitable from the point of view of saving costs [28]. The AFPRES-CLM study demonstrates the feasibility of collaborative practice to the extent that most of the pharmaceutical interventions requiring referral to a doctor were accepted.

Antihypertensive therapy substantially reduces the incidence of CVD, provided that BP is adequately controlled [29,30]. AFPRES-CLM showed an improvement in BP control similar to that observed in other studies [31,32,33]. In AFPRES-CLM this improvement was probably achieved thanks to the structured, individualized intervention of the pharmacist with each patient according to their particular needs (advice on lifestyle habits, ABPM, appropriate use of medications, etc.). This better BP control would lead to a reduction in CVR [34].

In the last few years, humanistic outcomes in clinical studies have become a major issue, as it is important to be aware of patients’ perceptions of their illness and treatment, the impact on their QL and how satisfied they are with the care they receive. In the AFPRES-CLM study we found no overall improvements in this outcome, which could be attributed to the asymptomatic and chronic nature of AHT, the treatment of which is not designed to relieve it, but to control it. These features of both the disease and its treatment make it difficult for patients to perceive possible improvements in their QL. Moreover, from the methodological point of view, the insufficient sample size, compared with what was initially anticipated, and the characteristics of the SF-36 generic measuring instrument, which is relatively insensitive to pharmaceutical care activities [35], could explain the absence of changes in respect of the patients’ QL. Some previous studies also failed to establish changes in the QL of hypertensive patients [36], in others, however, improvements were achieved through pharmaceutical intervention [37]. Such disparate results could be associated with the nature of AHT itself and with patients’ perception of it. However, in the pre–post comparison within each group we found no improvements in any of the dimensions of QL in the CG, but in the IG we did find statistically significant improvements in the physical pain and overall physical health dimensions, in line with other studies which have demonstrated that pharmaceutical intervention can significantly improve patients’ QL [38]. Curiously, several reports have shown that simplified treatment strategies improve the adherence to treatment and BP control in the elderly, meanwhile in diabetic adults with AHT, there is no better control [39,40].

Another result flowing from better BP control is a reduction in the use of healthcare resources, with the consequent cost savings for the healthcare system. Pharmaceutical care programs do not significantly increase the total direct costs of medical care, but we do see improvements in health outcomes for patients and in pharmaco-economic parameters [27,41,42].

AFPRES-CLM achieved a reduction in the use of healthcare resources, manifested in fewer visits to emergency departments, similarly to other studies [43]. However, no significant decreases were achieved in hospital admissions or in visits to primary care doctors or specialists, which have been demonstrated in other studies [44,45].

The Medication Review with Follow-Up (MRF) service provides clinical improvements in the study population and economic benefits arising primarily from a reduction in the use of healthcare resources; its widespread inclusion in pharmaceutical practice would therefore be desirable. This service contributes, in general, to rational use of medication, it is effective in optimizing pharmacotherapy and improving patients’ QL and it is also profitable for the healthcare system [46]. The number of MRPs is relatively high in patients with AHT, giving rise to poor BP control, among other adverse results, and it would therefore be advisable for MRF to be performed in these patients. The AFPRES-CLM study, similarly to other studies [47], shows how lack of adherence, suboptimal efficacy of medications and the need for additional monitoring of some patients are problems that could be associated with poor control of BP. It is important to note that adherence might be deeply affected not only by the pharmaceutical care, through pharmacotherapeutic monitoring that should increase this parameter, but also by how a concrete drug is well or not well tolerated by the patients, and this may push them to give up on therapeutic compliance. In AFPRES-CLM, 74.9% of MRPs were satisfactorily resolved, a higher result than in some previous studies [48,49] and lower than in others [50,51,52].

Poor chrono-pharmacotherapeutic adjustment is another of the factors causing lack of BP control in many patients. ABPM must be regarded as the new gold standard for diagnosing AHT and establishing the most appropriate individualized therapeutic regimen [53]. It also provides a more important value in predicting the risk associated with patients’ AHT, and it would therefore be advisable for ABPM to be performed on all patients with AHT [54]. Community pharmacies could be an appropriate environment for performing ABPM in collaboration with primary care doctors and specialists. The results of ABPM obtained in pharmacies are similar to those obtained by primary care doctors, and it is a more accessible setting for hypertensive patients [55], offering an opportunity to improve the quality of patient provision. In the AFPRES-CLM study ABPM measurements were performed on a group of susceptible patients, and this made it possible, in collaboration with the primary care doctor, to optimize their pharmacotherapy and achieve control of their BP, as in other pharmaceutical care programs that include ABPM [56,57]. Consideration should therefore be given to introducing it in pharmaceutical care services aimed at patients with AHT and CVR.

## 5. Conclusions

The APFRES-CLM community care program for hypertensive patients under pharmacological treatment is beneficial for controlling blood pressure in these patients and obtained an improvement in BP control in intervention patients (85.8% vs. 66.3%), as well as being effective in rationalizing the use of healthcare resources by reducing visits to emergency departments. A tendency can also be detected towards improving certain physical components of quality of life, and these need to be evaluated in future studies.

## Figures and Tables

**Table 1:** Initial characteristics of the sample relative to the main objective of the AFPRES-CLM study.

	Total (n = 388) 100%	Control Group (n = 202) 52.1%	Intervention Group (n = 186) 47.9%	*p*-Value
Gender—n (%)				
Male	174 (44.8%)	91 (45%)	83 (44.6%)	
Female	214 (55.2%)	111 (55%)	103 (55.4%)	1.000
Mean age—years ± SD	67.36 ± 11.5	68.52 ± 11.4	66.10 ± 11.5	0.035 *
Educational attainment—n (%)				
Higher education	50 (12.9%)	30 (14.9%)	20 (10.8%)	
Secondary	54 (13.9%)	22 (10.9%)	32 (17.2%)	
Primary	196 (50.5%)	102 (50.5%)	94 (50.5%)	
None	88 (22.7%)	48 (23.8%)	40 (21.5%)	0.235
Family history of AHT—n (%)				
No	185 (47.7%)	95 (47%)	90 (48.4%)	
Yes	203 (52.3%)	107 (53%)	96 (51.6%)	0.868
AHT complications—n (%)				
No	279 (71.9%)	142(70.3%)	137 (71.9%)	
Yes	109 (28.1%)	60 (29.7%)	49 (28.1%)	0.702
Diet recommended by healthcare professional—n (%)				
Yes	229 (59%)	123 (60.9%)	106 (57%)	
No	159 (41.0%)	79 (39.1%)	80 (43.0%)	0.489
Smoking—n (%)				
No (%)	348 (89.7%)	183 (90.6%)	165 (88.7%)	
Yes (%)	40 (10.3%)	19 (9.4%)	21 (11.3%)	0.658
Alcohol—n (%)				
No (%)	376 (96.9%)	198 (98.0%)	178 (957%)	
Yes (%)	12 (3.1%)	4 (2.0%)	8 (4.3%)	0.305
Physical activity—n (%)				
Intense	115 (29.6%)	64 (31.7%)	51 (27.4%)	
Moderate	115 (29.6%)	55 (27.2%)	60 (32.3%)	
None	158 (40.7%)	83 (41.1%)	75 (40.3%)	0.488
Hospital admission in the last 6 months—n (%)				
No	375 (96.6%)	195 (96.5%)	180 (96.8%)	
Yes	13 (3.4%)	7 (3.5%)	6 (3.2%)	1.000
Visit to emergency service in the last 6 months—n (%)				
No	338 (87.1%)	172 (85.1%)	166 (89.2%)	
Yes	50 (12.9%)	30 (14.9%)	20 (10.8%)	0.293
Visits to specialist—n (%)				
No	310 (79.9%)	163 (80.7%)	147 (79.0%)	
Yes	78 (20.1%)	39 (19.3%)	39 (21.0%)	0.779
Visits to primary care doctor—n (%)				
No	213 (54.9%)	112 (55.4%)	101 (54.3%)	
Yes	175 (45.1%)	90 (44.6%)	85 (45.7%)	0.901
BP control—n (%)				
Controlled	168 (46.4%%)	87 (47.0%)	75 (42.4%)	
Not controlled	194 (53.6%)	98 (53.0%)	102 (57.6%)	0.433
Abdominal circumference—mean cm ± SD	101.9 ± 16.7	103.3 ± 18.1	100.5 ± 14.9	0.158
Body mass index— mean kg/m^2^ ± SD	29.1 ±16.7	29.2 ± 4.1	28.9 ± 5.9	0.158
Pharmacies—n (%)	63 (100%)	32 (50.8%)	31 (49.2%)	0.854

AHT: arterial hypertension; BP: blood pressure. * *p*-values < 0.05 were considered statistically significant.

**Table 2:** Blood pressure control before and after the AFPRES-CLM pharmaceutical intervention.

	Before Pharmaceutical Intervention			After Pharmaceutical Intervention		
BP	CG n (%)	IG n (%)	*p*-Value	CG n (%)	IG n (%)	*p*-Value
**Controlled**	87 (47.0%)	75 (42.4%)		118 (66.3%)	145 (85.8%)	
**Uncontrolled**	98 (53.0%)	102 (57.6%)	0.433	60 (33.7%)	24 (14.2%)	<0.001 **

** *p*-values < 0.01 were considered statistically significant.

**Table 3:** Before–after comparison of quality of life measured with the SF-36 questionnaire relative to the main objective of the study.

	Control Group			Intervention Group		
	Start Mean ± SD	End Mean ± SD	Difference	Start Mean ± SD	End Mean ± SD	Difference
Physical function	75.8 ± 23.2	74.9 ± 22.9	–0.9	76.2 ± 24.0	77.7 ± 22.6	1.5
Physical role	80.6 ± 23.4	79.0 ± 24.3	–1.6	78.0 ± 25.7	80.3 ± 25.	2.3
Physical pain	29.7 ± 25.0	30.8 ± 25.7	1.1	34.7 ± 26.6	27.4 ± 24.47	–7.3
General health	58.4 ± 13.0	57.1 ± 12.9	–1.3	57.3 ± 11.1	56.1 ± 11.7	–1.2
Vitality	48.1 ± 15.0	53.1 ± 13.2	5.0	50.1 ± 16.1	53.5 ± 12.5	3.4
Social function	48.3 ± 14.2	48.4 ± 13.4	0.1	47.8 ± 12.2	48.3 ± 13.14	0.5
Emotional role	87.2 ± 20.5	84.9 ± 22.8	–2.3	85.2 ± 24.4	87.9 ± 21.1	2.7
Mental health	58.6 ± 10.1	57.9 ± 10.9	–0.7	58.1 ± 11.5	59.43 ± 10.45	1.3
Overall physical health	43.5 ± 5.6	45.9 ± 5.0	2.6	43.9 ± 5.4	42.9 ± 5.19	–1.0
Overall mental health	42.6 ± 5.9	42.7 ± 6.4	0.1	42.2 ± 6.4	43.6 ± 6.0	1.4

**Table 4:** Use of healthcare resources relative to achievement of the main objective of the AFPRES-CLM study.

	Before Pharmaceutical Intervention			After Pharmaceutical Intervention		
	GC n (%)	GI n (%)	*p*-Value	GC n (%)	GI n (%)	*p*-Value
Hospital admissions in the last 6 months due to AHT						
No	195 (96.5%)	180 (96.8%)		191 (97%)	175 (98.3%)	
Yes	7 (3.5%)	6 (3.2%)	1.000	6 (3%)	3 (1.7%)	0.508
Visits to emergency department in the last 6 months due to AHT						
No	172 (85.1%)	66 (89.2%)		179 (90.9%)	173 (97.2%)	
Yes	30 (14.9%)	20 (10.8%)	0.293	18 (9.1%)	5 (2.8%)	0.002 **
Visits to specialist in the last six months due to AHT						
No	163 (80.7%)	147 (79.0%)		160 (81.2%)	142 (79.8%)	
Yes	39 (19.3%)	39 (21.0%)	0.779	37 (18.8%)	36 (20.2%)	0.824
Visits to primary care physicians in the last six months due to AHT						
No	112 (55.4%)	101 (54.3%)		113 (57.4%)	103 (57.9%)	
Yes	90 (44.6%)	85 (45.7%)	0.901	84 (42.6%)	75 (42.1%)	1.000

** *p*-values < 0.01 were considered statistically significant.

**Table 5:** Distribution of MRPs detected in hypertensive patients included in the AFPRES-CLM study.

Medication-Related Problems	Number
Self-medication	17
Unnecessary medication	3
Untreated health problem	44
Non-adherence	65
Insufficiently treated health problem	104
Inappropriate dose, schedule, dosage	46
Interaction	5
Probability of adverse effects	67
Personal characteristics	3
Total	354

## Data Availability

The authors declare that the data that support the findings of this study are available from the corresponding author upon reasonable request.

## Abbreviations

Arterial hypertension (AHT); Cardiovascular risk factors (CVRFs); Blood pressure (BP); Cardiovascular disease (CVD); Cardiovascular risk (CVR); Systolic Blood Pressure (SBP); Diastolic Blood Pressure (DBP); Control Group (CG); Intervention Group (IG); Pharmacotherapeutic Monitoring (PTM); Medication-Related Problems (MRPs); Negative Outcomes associated with Medication (NOMs); Ambulatory Blood Pressure Monitoring (ABPM); Quality of life (QL); Medication Review with Follow-Up (MRF).
